# Ambulatory EEG findings in patients with neurodegenerative disease

**DOI:** 10.1002/alz70857_103311

**Published:** 2025-12-25

**Authors:** Stephanie Tran, Niyatee Samudra

**Affiliations:** ^1^ Stanford, Palo Alto, CA, USA; ^2^ Stanford University, Palo Alto, CA, USA

## Abstract

**Background:**

The prevalence of subtle or subclinical seizures and interictal epileptiform discharges is being increasingly recognized in patients with neurodegenerative disease. These patients can also frequently experience episodic altered awareness or cognitive fluctuations of unclear etiology, even in the absence of known history of epilepsy. Accordingly, electroencephalographic findings in this population are of interest. Ambulatory EEGs are often ordered in patients with these symptoms. They have prolonged duration compared to routine EEGs, and these studies can be expected to increase the yield for epileptiform findings. They have the potential to change clinical management in patients with neurodegenerative disease. In addition, indications to obtain ambulatory EEGs in patients with neurodegenerative disorders are not well established.

**Method:**

A single‐center retrospective analysis of ambulatory EEGs between January 2023 and December 2024, ordered in patients over 45 (approximately 450 studies), who are found to have a diagnosis of mild cognitive impairment (MCI) or dementia on chart review, will be conducted. Demographic and medical information, as well as pertinent neurologic or imaging diagnoses, will be abstracted from charts for patients with a diagnosis of MCI or dementia. Suspected neurodegenerative pathology will be established. In addition, indications for ambulatory EEG, ambulatory EEG duration, EEG abnormalities, and whether the study appeared to change management will be ascertained. Predictors of epileptiform abnormalities as well as whether the study changed management will be evaluated.

**Result:**

The results from this retrospective study are expected to provide valuable information regarding the yield of ambulatory EEG in patients with neurodegenerative disease.

**Conclusion:**

Ultimately, this and similar studies may provide guidance regarding guidelines for the use of prolonged EEG in this population, particularly in those patients who experience fluctuations without clear history of seizure, as indications are currently unclear.

